# Microwave Treatment Improves Coarse Cereals' Texture and Cooking Quality

**DOI:** 10.1002/fsn3.71082

**Published:** 2025-10-22

**Authors:** Hong Song, Dingyi Li, Hongyu Zhang, Lina Yang, Danshi Zhu, Hongliang Fan, Lu Han, Jinxin Li, He Liu

**Affiliations:** ^1^ College of Food Science and Technology Bohai University Jinzhou China

**Keywords:** coarse cereals, gelatinization, microwave power, pretreatment

## Abstract

Use of microwave heating has increased in the coarse cereals sector due to its versatility. In order to improve the poor cooking quality and rough mouthfeel of coarse cereals, the effects of microwave treatment on the cooking quality of soaked coarse cereals and sensory score of cooked coarse cereals were studied, and the improvement mechanism was revealed from the aspects of water distribution, starch ordered structure, and microstructure. The microwave treatment significantly improved the quality of coarse cereals. Especially after 480 W microwave treatment for 10 min, the hardness and chewability of coarse cereals decreased from 3162.63 and 378.12 N to 2039.15 and 133.13 N, respectively. The water fluidity was improved and part of the immobile water was transformed into tightly bound water. The microwave treatment promoted a reduction in the values of peak viscosity, final viscosity, breakdown, and setback for samples. Microwave treatment increased the short‐range ordered structure of starch, and scanning electron microscopy showed that the surface of starch particles was rough, with increased cracks and pores. In addition, microwave treatment significantly improved coarse cereals eating quality. The cooking time was significantly shortened, allowing it to be cooked with rice. The texture properties (e.g., hardness, cohesiveness, and chewiness) and sensory properties of cooked coarse cereals were significantly ameliorated by microwave treatment. This study helps to provide a new way for producing high‐quality coarse cereals.

## Graphical abstract

Microwave treatment improved the comprehensive utilization of brown rice, mung beans, black beans, cowpeas, chickpeas, etc. Microwave treatment improved the cooking quality and eating quality of coarse cereals. Microwave treatment improved the fluidity of water in coarse cereals. The relative crystallinity of coarse cereals was reduced by microwave treatment. Surface roughness and internal cracks of starch particles occurred after treatment.

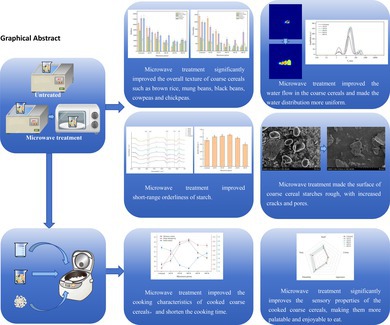

## 1 Introduction

The global consumption of instant and rapid cooking meals has surged. Coarse cereals are hard in structure and difficult to cook, and do not meet the need of today's fast‐paced lifestyle for food consumption, limiting their applicability in convenient coarse cereals food (Sun et al. 2023). Brown rice, obtained from rice grains after hulling, retains bran and embryo, accumulating 60%–70% of the non‐starch nutrients of rice, rich in polyphenols, dietary fiber, and other substances, thus possessing higher nutritional value compared to white rice (Saleh et al. 2019). In recent years, whole grain represented by brown rice has attracted increasing attention, and has become a new generation of dietary staple food widely promoted all over the world. Pulses (mung beans, black beans, cowpeas, and chickpeas) are common examples of difficult‐to‐cook miscellaneous grains that are often used in daily life. They are well known for their favorable nutritional profiles, which include relatively high levels of proteins, dietary fibers, vitamins, and phytochemicals and low contents of rapidly digestible starch and lipid (except for chickpeas) (Ren et al. 2020). Brown rice and pulses represent a good source of staple food. They can offer rich sensory, such as brown rice provides chewiness, mung beans, cowpeas, and chickpeas adding softness and stickiness, and black beans enhancing the layers. In addition, these coarse cereals (brown rice, mung beans, black beans, cowpeas, and chickpeas) are rich in essential amino acids that are lacking in rice. Eating them together with rice can achieve a nutritional supplement effect. The glycemic index of coarse cereals is low, which can slow the rate of starch absorption by the human body, and is often used as a diet therapy for metabolic diseases such as diabetes, hyperlipidemia, and hyperglycemia (Liu et al. 2021; Olagunju 2022).

As well as being rich in nutrients, the fiber layer on the coarse cereals' surface is closely arranged, which hinders the infiltration of water and heat transfer during cooking, making it challenging to gelatinize and increasing the cooking time. In addition, the cellulose content directly affects the taste, digestion, and absorption of coarse cereals, which reduces people's willingness to consume them. As a result, the degree of staple food use of coarse cereals is low. To tackle this issue, there is an increasing demand to develop an efficient method to pretreat coarse cereals to improve their cooking efficiency and quality.

The traditional method of processing coarse cereals has problems such as long processing cycles and significant loss of nutritional components, making it difficult to meet modern people's needs for healthy, nutritious, and convenient food. Microwave heating is a common food heating method in daily life, which has strong penetration. Microwave technology is also used in coarse cereals pretreatment. Najib et al. (2023) reported the substitutability of conventional thermal treatments (roasting and convective air drying) with greener microwave‐based technologies (microwave, microwave‐infrared, and vacuum‐microwave drying) with a similar range of temperatures to encourage the food industry to use these novel efficient thermal treatments. The performance of these techniques was assessed based on the level of starch gelatinization and changes in its structural, morphological, nutritional, and functional properties in the drying process of soaked and germinated lentils. Cao et al. (2022) discovered that the texture characteristics of quinoa first increased and then decreased as microwave treatment time extended. The alternating electromagnetic field of microwaves destroyed the original vesicular aggregation structure of starch particles, causing the starch to disperse into separate particles from its aggregated state. However, prolonged high‐intensity microwave treatment could lead to partial gelatinization of quinoa starch, forming a rigid structure. These changes impacted the gelatinization and thermodynamic properties of quinoa starch, although there was no difference in gelatinization temperature. Microwaves can affect the rotational state of the starch's double‐helix structure, resulting in fluctuations in starch crystallinity. Additionally, the microwaves' alternating electric field could modify certain chemical bonds on amylopectin branches, making the starch more likely to combine with water to form an ordered gel network structure. This was beneficial for enhancing quinoa quality. Bai et al. (2021) reported that microwave treatment of highland barley caused the structure of outer layers to compress and deform, leading to the formation of numerous cavities and disruption of the endosperm cell structure. This significantly increased the content of free phenolics and extractability of β‐glucan. Therefore, microwave cooking can be a rapid alternative to retain coarse cereals' quality and nutritional value (Chin et al. 2020). Although these results have been published in some cereals or pulses, it is effective in improving the texture as well as cooking quality and other properties by microwave treatment. However, the limitation of these studies was that only one type of crop—either cereal or pulse within one paper. Research on microwave pre‐cooked five coarse cereals is rarely studied in the world, and limited study is available on water distribution, starch ordered structure, and microstructure during gelatinization. A standardized protocol was necessary to ensure compatibility between the coarse cereals and rice to prepare varieties of mixed coarse cereals products.

To fill this identified research gap, our study effectively utilized the advantages of microwaves and deeply analyzed the changes in cooking and eating quality of five coarse cereals after microwave pre‐cooking. This comprehensive approach has enabled us to gain a deeper understanding of the mechanisms by which microwave pretreatment enhances the quality of coarse cereals and to identify optimal treatment conditions for each type. Performance was assessed based on the level of coarse cereals' changes in its texture properties, water distribution, gelatinization degree, crystallinity, short‐range order of starch, and microstructure using various analytical techniques, including low‐field nuclear magnetic resonance (LF‐NMR), X‐ray diffraction (XRD), scanning electron microscopy (SEM), and Fourier transform infrared spectroscopy (FTIR). Using brown rice, mung beans, black beans, cowpeas, and chickpeas as the main raw ingredients, a type of precooked coarse cereals suitable for cooking together with rice was developed, providing the technical basis for the research and development of nutritious, healthy, and convenient coarse cereals food.

## 2 Materials and Methods

### 2.1 Materials

The raw materials of brown rice, mung beans, black beans, cowpeas, and chickpeas used were purchased from Beipiao Yongfeng Cereals Co. Ltd., Chaoyang, Liaoning Province, China.

### 2.2 Single Factor Test of Pretreatment of Coarse Cereals

10 g of each type of coarse cereal were weighed out and mixed with water at a material‐to‐liquid ratio of 1:2.5, which was soaked for 2 h at a constant temperature of 40°C. The soaked coarse cereals (50 g) were treated by microwave oven (MKJ‐JIN, Qingdao Microwave Innovation Technology Co. Ltd., Qingdao, Shandong) with different power (160, 320, 480, 640, and 800 W) for different time (6, 8, 10, 12, and 14 min). The degree of starch gelatinization in coarse cereals was evaluated using enzymatic hydrolysis.

#### 2.2.1 Observation of Surface Morphology

Images of treated coarse cereals were collected using a high‐performance digital single‐lens reflex camera (EOS 70D, Canon Corporation, Tokyo, Japan). Photographs of samples were taken in a small photo studio (Sutefoto, Shenzhen Sute Photographic Equipment Co. Ltd., Shenzhen, Guandong, China).

#### 2.2.2 Textural Properties

A total of 2 g of microwave pretreatment samples and cooked samples were cooled to room temperature for 1 h for testing, respectively. The textural properties were measured using a Texture Analyzer (TA‐XF plus, Bosin Tech., Shanghai, China) with a 50‐mm cylindrical probe, and the speeds of pre‐test, test, and pro‐test were 1, 0.5, and 1 mm/s, respectively. A two‐cycle compression with 0.049 N induction force and 70% compression force was performed for texture property analysis. The texture parameters (hardness, adhesiveness, springiness, cohesiveness, gumminess, and chewiness) were then calculated from the test curves. Each test was repeated eight times and the mean value recorded (Xiong et al. 2023).

#### 2.2.3 Determination of Moisture Binding State

The proton relaxation time measurements were performed using a previously reported method with a 23 MHz LF‐NMR analyzer (MesoMR23‐040H‐I, Niumag Co. Ltd., Shanghai, China) to observe hydrogen proton migration under different microwave treatment conditions. About 6.0 g of sample was placed in a 40‐mm diameter cylindrical glass tube supplied with the instrument to prevent evaporation. The untreated sample was directly dosed into the glass tube. The transverse relaxation time (T_2_) of the sample was measured using a Carr–Purcell–Meiboom–Gill (CPMG) pulse sequence. T‐invfit software was used to fit the data to get the multicomponent relaxation spectrum (T_2_) of the samples and determine the relaxation time of each characteristic peak (T_21_, T_22_, T_23_…, T_2i_) and the corresponding peak area ratio (M_21_, M_22_, M_23_…, M_2i_, etc.). The main operating parameters follow: number of sampling points 74,992, number of echoes 2500, echo time 0.3 ms, attenuation relaxation time 4000 ms, and cumulative number 4 (Xiong et al. 2023). All were operated at a temperature of 25°C.

#### 2.2.4 Observation of Water Distribution

Imaging software (Version 1.06, Niumag Analytical Instrument Corporation, Suzhou, China) was used to collect two‐dimensional magnetic resonance images on an MRI (Magnetic Resonance Imaging) analyzer. Spin echo imaging sequences were used to obtain T_2_ images. The scanning protocols follow: mean 2, read size 256, and phase size 192. Echo and repeat times were 50 and 1900 ms, respectively. The signal strength was measured and analyzed using the free and open‐source software Osirix (Version 1.06, Niumag NMR Date Analysis Software, Suzhou, China).

#### 2.2.5 Gelatinization Properties

The gelatinization properties of samples were determined by RVA‐4500 (PerkinElmer, Sydney; Australia) following the method of Qi et al. (2020) with modifications as follows. The samples were dry ground (3 g, 12% moisture base), and 25 g of distilled water was placed in the RVA chamber. Pretreatment of coarse cereal powder paste involved maintaining at 50°C for 1 min, heating at 12°C/min to 95°C, and holding at 95°C for 2.5 min. Finally, the temperature was lowered to 50°C at 12°C/min and held there for 2 min. The coarse cereal powder was first mixed at 960 rpm for 10 s to fully disperse it; then, the paddle speed was set to 160 rpm for the rest of the run (Xing et al. 2023).

#### 2.2.6 XRD Detection

Crystalline structures of pretreated coarse cereals samples were analyzed using XRD (D8 ADVANCE, Bruker GmbH, Germany), conducted at 40 mA and 40 kV, using Cu Kα radiation at a wavelength of 0.1542 nm. The scanning range of the diffraction angle (2θ) was 5°–45° at a speed of 6°/min and a step size of 0.05° (Han et al. 2021). The scan results were analyzed using the software Jade (version 5.0).

#### 2.2.7 FTIR Detection

The freeze‐dried coarse cereals sample was ground and mixed with potassium bromide (KBr). The mixture was placed in a KBr pellet container. Spectra were recorded using an FTIR spectrometer (Bruker Alpha, Germany) by scanning samples at wavelengths 4000–400 cm^−1^. The spectrum was analyzed by monitoring changes in various chemical entities within the sample (Govindaraju et al. 2022).

#### 2.2.8 SEM Detection

The microwave‐pretreated chickpeas, cowpeas, mung beans, black beans, and brown rice were mixed in equal proportions, ground into powder, and passed through 100 meshes. The particle surface of coarse cereals powder was detected by SEM (S‐4800, Hitachi, Japan) at 10 kV acceleration potential. The powders were fixed on the sample stage with conductive adhesive and then sputter‐coated with gold before determination (Maaran et al. 2014).

### 2.3 Application of Pretreated Coarse Cereals

#### 2.3.1 Preparation of Cooked Coarse Cereals and Rice

Equal amounts of pretreated coarse cereals and rice was accurately weighed in the container, and 2.5 times the amount of water to that of all the weight was added for the cooking process. Pretreated coarse cereals and rice was cooked in an electronic rice cooker (Supor, model CFXB20FC17‐35, Zhejiang, China) by heating for 30 min, and cooled to room temperature (25°C) in preparation for measuring the sensory evaluation and textural properties (Ren et al. 2020).

#### 2.3.2 Sensory Evaluation

The sensory evaluation of cooked coarse cereals was conducted according to Dang et al. (2018). Cooked coarse cereals were placed in white dishes and randomly coded with three digits. The samples were randomly served to trained panelists (five male and five female, aged 20–30 years) in a room temperature environment. The total sensory score was 100 points: odor 20 points, appearance 20 points, palatability 30 points, flavor 25 points, and cold texture 5 points. The evaluators should perform oral cleaning before each test. A 2‐min rest was enforced between sample evaluations to minimize any carryover effect (Muroki et al. 2024).

#### 2.3.3 Measuring Moisture Content

The coarse cereals pretreated using different microwave powers were stored in a sealed plastic bag. After all drying tests, the moisture content of cooked coarse cereals was measured immediately according to AOAC method. Each measurement was repeated three times and the results reported as mean ± standard deviation (Shen et al. 2021).

#### 2.3.4 Water Absorption

Precisely weighing 20 g of coarse cereals, which were soaked for 2 h and precooked with different microwave power (0, 160, 320, 480, 640, and 800 W) for 10 min. Then 200 mL of distilled water was added for further steaming. The cooked samples were drained and weighed to calculate the amount of water absorbed during cooking with the equation below (Huang et al. 2024):

(1) $$\text{Water absorption}\%=\frac{m_{1}-m_{0}}{m_{0}}\times 100\%$$

where *m*_0_ is mass of dry weight of the coarse cereals and m_l_ is the mass of the cooked coarse cereals drained.

#### 2.3.5 Solid Content

The solid content in cooked coarse cereals was evaluated according to a previous report (Chen et al. 2022). After cooking, solid content was determined using a 10 g sample subjected to oven drying at 105°C for 24 h.

(2) $$\text{Solid content}\%=\frac{w_{0}}{w_{1}}\times 100\%$$

where *W*_0_ is dry material weight and *W*_1_ is wet material weight.

### 2.4 Statistical Analysis

Statistical analyses were carried out using SPSS version 27.0. To evaluate the difference between means, one‐way ANOVA was used followed by Duncan's test. Significance was defined as *p* < 0.05. Sigmoid fitting was performed with Origin 2023 (Li et al. 2015).

## 3 Results and Discussion

### 3.1 Effect of Pretreatment on the Gelatinization Degree of Coarse Cereals

The water in soaking is expected to diffuse evenly into the seeds. In this situation, the moisture content after soaking inside the seeds is a crucial factor in the extent of starch gelatinization. Previous studies reported that when the seed moisture exceeds 30%, the husks split, deforming the kernels. Therefore, long time soaking needed to be stopped when the moisture content of the seed reached ≈30% (Han JungAh and Lim SeungTaik 2009). The initial moisture content and the moisture content after soaking are presented in Table 1. It is evident that soaking time has a significant influence on the moisture content of five coarse cereals. As the soaking duration increases to 2 h, the moisture content of all samples gradually rises. The moisture content of chickpeas increased steadily from 3.15% at 0 min to 26.82% at 120 min. The moisture content of cowpea increased from 1.96% to 28.02%, and the moisture content increased significantly after soaking for 60 min. The moisture content of mung bean increased slowly from 1.64% to 2.96% from 0 to 60 min, and increased rapidly from 2.96% to 24.53% from 60 to 120 min. The moisture content of black bean increased significantly from 1.19% to 28.71%. The moisture content of brown rice increased relatively steadily from 3.07% to 26.05%. In conclusion, all coarse cereals exhibited varying degrees of enhanced water absorption capacity during the soaking process, with black beans and cowpeas demonstrating the most pronounced increases in moisture content. These processes can alter the permeability of the cell wall, and water interacts with various components within coarse cereals.

**TABLE 1:** Effects of different pretreatments on gelatinization degree of five coarse cereals before and after cooking with rice.

Sample	Treatments	Content				
		Chickpeas	Cowpea	Mung bean	Black bean	Brown rice
Moisture content (%)						
Soaking	0 h	3.15 ± 0.29^a^	1.96 ± 0.11^a^	1.64 ± 0.61^a^	1.19 ± 0.31^a^	3.07 ± 0.87^a^
	1 h	19.41 ± 1.37^b^	6.12 ± 0.58^b^	2.96 ± 0.49^a^	16.80 ± 0.94^b^	13.54 ± 0.85^b^
	2 h	26.82 ± 0.73^c^	28.02 ± 0.90^c^	24.53 ± 1.23^b^	28.71 ± 1.32^c^	26.05 ± 0.35^c^

		Chickpeas	Cowpea	Mung bean	Black bean	Brown rice
Gelatinization degree (%)						
Before cooking	Microwave power					
	160 W	35.44 ± 2.05^e^	38.59 ± 2.39d	29.30 ± 2.24^e^	35.51 ± 2.18^d^	37.46 ± 2.17^e^
	320 W	49.55 ± 2.42^d^	48.25 ± 2.24^b^	48.98 ± 2.49^d^	50.33 ± 1.98^c^	54.72 ± 2.05^d^
	480 W	59.55 ± 2.13^c^	60.15 ± 1.99^c^	63.03 ± 2.31^c^	65.39 ± 2.18^b^	64.44 ± 2.01^c^
	640 W	65.67 ± 1.61^b^	69.14 ± 2.13^a^	69.27 ± 2.13^b^	67.49 ± 2.08^ab^	69.38 ± 2.06^b^
	800 W	70.16 ± 1.98^a^	71.89 ± 2.53^a^	76.56 ± 2.21^a^	70.45 ± 2.02^a^	74.43 ± 2.11^a^
	Microwave time					
	6 min	39.19 ± 2.01^d^	51.26 ± 1.97^d^	49.23 ± 2.02^c^	49.58 ± 2.11^d^	56.45 ± 1.94^c^
	8 min	48.50 ± 2.27^c^	55.14 ± 2.05^c^	59.42 ± 2.38^b^	58.60 ± 2.16^c^	57.43 ± 2.43^c^
	10 min	60.51 ± 2.40^b^	61.17 ± 2.05^b^	61.75 ± 1.77^b^	60.58 ± 2.10^b^	60.65 ± 2.05^b^
	12 min	65.15 ± 2.01^a^	69.68 ± 2.05^a^	64.69 ± 1.97^a^	63.51 ± 1.85^a^	62.43 ± 1.68^b^
	14 min	69.15 ± 2.56^a^	73.18 ± 2.02^a^	66.54 ± 2.15^a^	65.47 ± 1.94^a^	70.50 ± 1.81^a^
After cooking	Microwave power					
	160 W	53.44 ± 1.05^d^	56.59 ± 1.32^e^	47.3 ± 1.82^e^	53.51 ± 1.11^d^	51.46 ± 1.41^e^
	320 W	69.55 ± 1.35^c^	68.25 ± 1.65^d^	64.98 ± 1.19^d^	66.33 ± 1.43^c^	70.72 ± 1.38^d^
	480 W	85.55 ± 2.15^b^	86.15 ± 1.44^c^	85.03 ± 2.01^c^	87.39 ± 1.85^b^	85.44 ± 2.01^c^
	640 W	89.67 ± 2.03^a^	90.14 ± 1.56^b^	90.27 ± 1.42^b^	89.49 ± 1.32^b^	89.38 ± 1.56^b^
	800 W	92.16 ± 2.16^a^	93.89 ± 1.57^a^	93.56 ± 1.46^a^	92.45 ± 1.67^a^	92.43 ± 1.40^a^
	Microwave time					
	6 min	57.19 ± 2.05^d^	69.26 ± 2.01^d^	67.23 ± 2.01^d^	67.58 ± 1.75^d^	70.45 ± 1.59^d^
	8 min	68.5 ± 1.85^c^	75.14 ± 2.05^c^	75.42 ± 2.12^c^	74.6 ± 1.82^c^	73.43 ± 2.01^d^
	10 min	86.51 ± 1.6^b^	87.17 ± 1.67^b^	85.25 ± 1.85^b^	85.58 ± 2.11^b^	85.15 ± 1.48^c^
	12 min	89.15 ± 1.89^ab^	91.68 ± 1.34^a^	88.69 ± 1.72^ab^	89.51 ± 1.78^a^	88.43 ± 1.80^b^
	14 min	91.15 ± 1.49^a^	93.18 ± 1.56^a^	91.54 ± 2.00^a^	91.47 ± 1.68^a^	92.5 ± 1.50^a^

*Note:* values with different letters in the same column are significantly different (*p* < 0.05).

According to a previous study reported (Guo et al. 2022; Zhu et al. 2023), coarse cereals can be co‐cooked with rice when their gelatinization degree reaches 85% after pretreatment. Excessive gelatinization of coarse cereals can cause loss of nutrition and sensory perception. Table 1 reveals that all five tested coarse cereals (chickpea, cowpea, mung bean, black bean, and brown rice) consistently demonstrated enhanced gelatinization degree with increasing microwave power and time. However, all gelatinization degrees were less than 85%. After pretreatment, the coarse cereals cooked with rice, their gelatinization degrees were measured. As shown in Table 1, the pretreatment significantly improved the gelatinization degrees of all five types of coarse cereals. After 480 W for 10 min of microwave pretreatment, the gelatinization degrees of chickpeas, cowpea, mung bean, black bean, and brown rice were 85.55%, 86.15%, 85.03%, 87.39%, and 85.44%, respectively, which were above the national standard for direct cooking gelatinization degrees of rice chaff foods. Therefore, chickpeas, cowpea, mung bean, black bean, and brown rice can be cooked together with rice after 480 W for 10 min of microwave pretreatment.

### 3.2 Surface Morphological Structures of Microwave‐Treated Coarse Cereals

**FIGURE 1:**
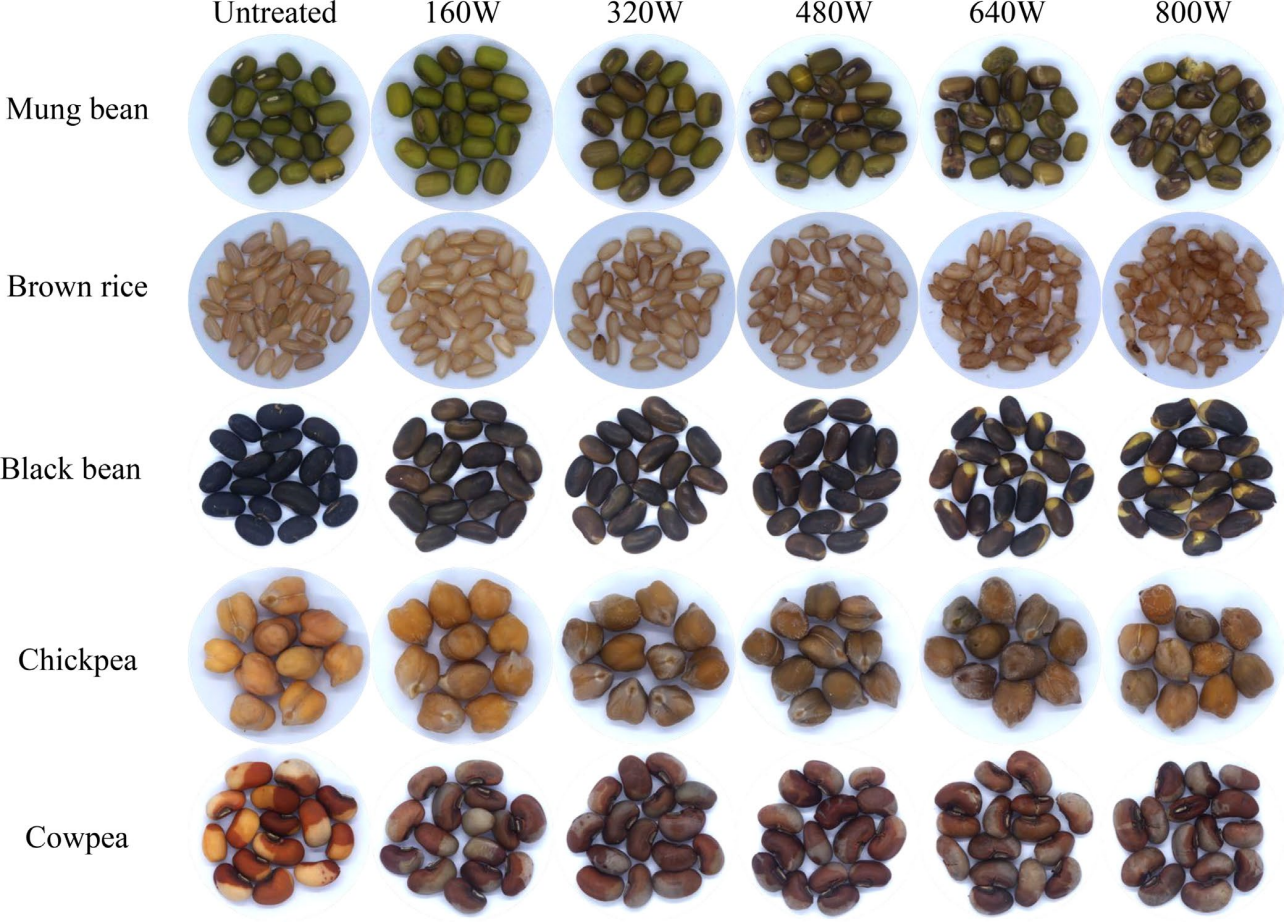
Apparent structural changes of coarse cereals after different microwave treatments (160, 320, 480, 640, or 800 W for 10 min).

Appearance of untreated and microwave‐treated coarse cereals for different microwave power conditions is shown in Figure 1. After being subjected to microwave power of 480–800 W, the surfaces of treated mung beans and black beans exhibited cracks to varying degrees. These might be because the high‐temperature condition caused “flash off” of inner liquid water to vapor, and this sudden evaporation resulted in pressure generation within the seeds and caused them to expand (Bai et al. 2021). After 800 W microwave treatment for 10 min, the surface cracks of mung beans and black beans increased, and their color notably deepened. Similarly, the variation in color of microwave‐treated brown rice, cowpea, and chickpea also increased. This color change is likely attributed to the Maillard reaction and caramelization processes, which are accelerated under high‐temperature conditions. These reactions occur between reducing sugars and amino acids or proteins present in the seeds, leading to the formation of melanoidins and other complex compounds; this is consistent with what Gökmen et al. (2008) reported. Similarly, microwave treatment of brown rice, cowpea, and chickpea also resulted in increased color variation, suggesting that water vaporization‐induced pressure buildup and subsequent thermal degradation reactions are at play in these coarse cereals.

### 3.3 Texture Properties of Microwave‐Treated Coarse Cereals

**FIGURE 2:**
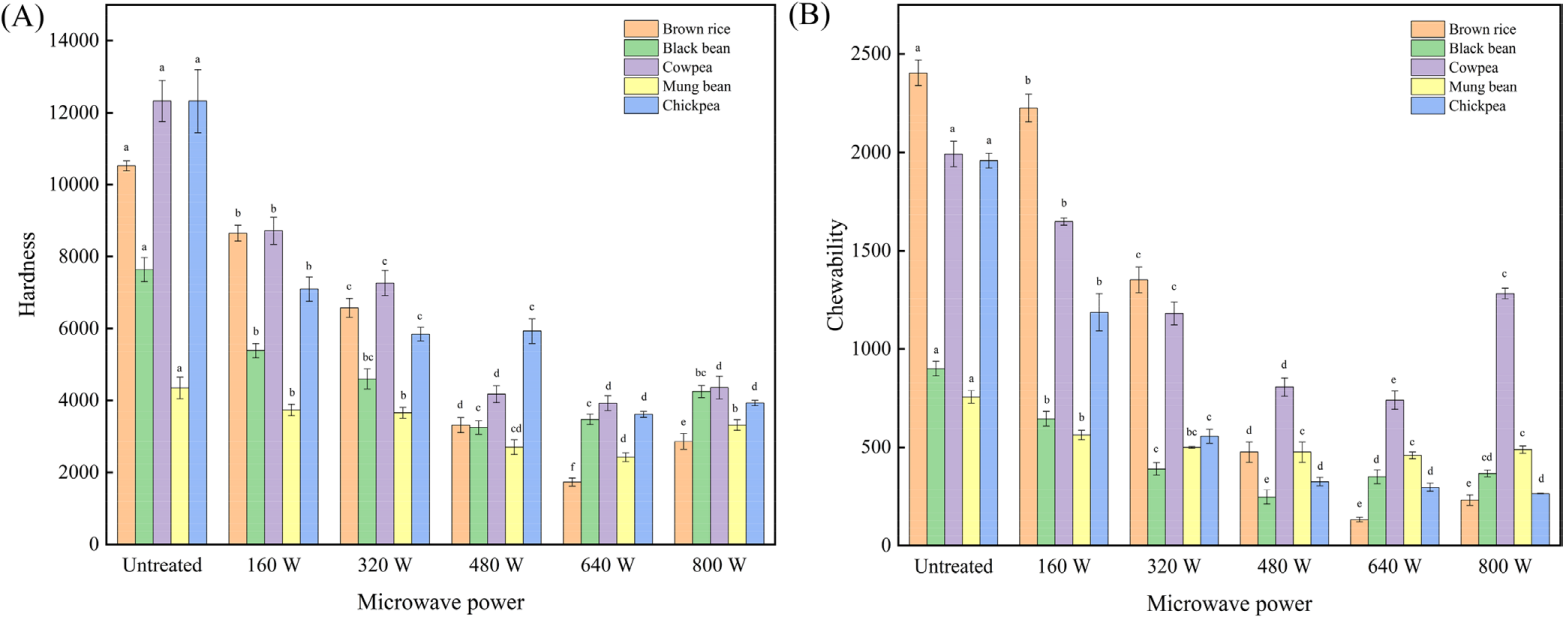
Changes of hardness (A) and chewiness (B) of coarse cereals after microwave treatment.

The effects of microwave power on the texture of five kinds of coarse cereals are shown in Figure 2. Compared to untreated coarse cereals, the hardness and chewiness of microwave‐treated coarse cereals were significantly reduced. The hardness of black beans had a minimum value of 3251.04 N for microwave power of 480 W. The hardness of brown rice, cowpea, mung bean, and chickpea had minimum values for microwave power 640 W, with 1735.35, 3924.21, 2429.03, and 3614.4 N, respectively. Chewiness was positively correlated with hardness (Xing et al. 2023). For microwave power of 640 W, the minimum chewability values for brown rice, cowpea, and mung bean were 133.34, 738.79, and 458.05 N, respectively. Black beans had a minimum chewability of 246.66 N at 480 W, while chickpeas demonstrated a minimum chewability of 263.94 N at 800 W.

Microwave‐treated coarse cereals samples had lower hardness values than control samples. This also implied that microwave treatment damaged coarse cereals' structure, thus allowing water to enter the brown rice more quickly, causing starch pasting and reducing its hardness and chewiness (Soltani et al. 2023). The microwave power > 480 W caused cracks on the surface of coarse cereals (Figure 1), which helped water penetrate deep into the particles, resulting in increased amylose content and a tighter molecular structure of amylose, and led to increased hardness and chewiness of coarse cereals. This is consistent with the previous results (Hu et al. 2022; Li et al. 2024). Moreover, after 800 W treatment, coarse cereals' hardness tended to increase, possibly related to the loss of water and the change of internal microstructure of coarse cereals, and the expansion and rearrangement of starch particles would increase coarse cereals' hardness (Lin et al. 2023).

### 3.4 Water Distribution and Water Migration of Microwave‐Treated Coarse Cereals

**FIGURE 3:**
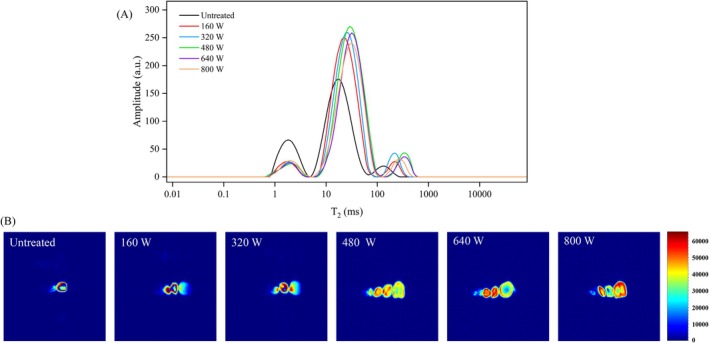
LF‐NMR transverse relaxation time (T_2_) distribution curves (A) and water distribution (B) of samples for different microwave power treatments.

The water distribution of coarse cereals after different microwave power treatments was studied by LF‐NMR and illustrated using transverse relaxation time (T_2_; Figure 3A). Numerous studies have shown an inverse relationship between T_2_ and the mobility of water molecules. Specifically, a shorter T_2_ corresponds to reduced mobility of water molecules, whereas a longer T_2_ is associated with increased mobility, making the water more susceptible to being expelled or lost (Su et al. 2020). According to T_2_ of samples, three water fractions were characterized by immobilized water, surface water, and free water in this work. The transverse time of all samples occurred at 0.9–5 ms (T_21_), around 5–88 ms (T_22_), and 88–1000 ms (T_23_), in accordance with immobilized water, surface water, and free water, respectively (Xiong et al. 2023). Compared with untreated coarse cereal samples, all microwave‐treated coarse cereal samples showed a significantly increased T_2_. This suggested that the fluidity of water molecules inside the sample increased (Li et al. 2022). It was clear that the semaphore of each component underwent alterations subsequent to microwave treatment, with a significant decrease in T_21_ and a pronounced increase in T_22_ and T_23_. This suggested that the increase in total relative water content was mainly due to surface water and free water.

The relaxation time of T_22_ water moved right when microwave power was < 480 W, and moved left when > 480 W. This meant that the fluidity of the less mobile water in coarse cereals increased and then decreased with increased microwave power. For microwave power of 800 W, the electromagnetic effect and heating effect of microwaves would lead to excessive gelatinization of coarse cereals, forming a rigid structure inside coarse cereals. The rigid structure would weaken the ability of binding water in the interstitium of coarse cereals during subsequent cooking (Cao et al. 2022).

Magnetic resonance imaging can display a proton density‐weighted image of the interior of the sample, reflecting the distribution of hydrogen protons in the coarse cereals sample. In general, a higher hydrogen proton density produces a brighter proton density‐weighted image. The proton density‐weighted image is generated by the magnetic resonance imaging analyzer into a grayscale image, which cannot clearly show water distribution inside the coarse cereals. Therefore, images were converted into false color images by nuclear magnetic software: the red area indicates the high hydrogen proton semaphore and the green area indicates the low hydrogen proton semaphore. This provides an intuitive and visual analysis of the water distribution inside samples (Li et al. 2020). The image of the raw coarse cereals was almost blue (Figure 3B), indicating that the untreated sample contained little free water and weakly bound water. Compared to the raw sample, the color changed significantly after microwave treatment. As microwave power increased, more red areas appeared on the proton density‐weighted images, and the water gradually penetrated the interior of the cotyledons, forming a water gradient in the cotyledons (Xiong et al. 2023). In samples pretreated at 480 W, the moisture content in the central part of the coarse cereals increased, the signal intensity of the edge of the coarse cereals and the central part tended to be consistent, the water ladder difference between the inner and outer layers was small, the water distribution was more uniform, and the starch on the coarse cereals surface was gelatinized to form a coating. The coating physically impedes the diffusion of water under high power conditions (An et al. 2024). With increased microwave power, the water diffusion rate in coarse cereals increased. For 640 and 800 W, the water diffusion of chickpea, cowpea, mung bean, and brown rice from the inside to the outside phenomenon, and hardness increased, consistent with the texture profile analysis.

### 3.5 Gelatinization Characteristics and Water Migration of Microwave‐Treated Coarse Cereals

The RVA was used to investigate the effect of microwave power on pasting properties of coarse cereals (Zhang et al. 2022). Below 480 W, peak viscosity (PV) decreased with increasing microwave power (Table 2). When the power reached 480 W, PV showed an upward trend, and the lowest value was 696.00 cP. The PV is affected by the degree of amylose leaching, particle expansion, and lipid–amylose complex (Joshi et al. 2013; Zheng et al. 2020). In general, high amylose content exhibits low PV (Reddy et al. 2017). However, when microwave power reached 640 W, PV again increased significantly, possibly related to the dual electromagnetic and thermal effects of microwaves. The trough viscosity (TV) of coarse cereals decreased with increased microwave power, indicating a reduction in starch viscosity after PV due to the breakdown of swollen granules by high temperature (Reddy et al. 2017), and ranged within 683.67–1548.67 cP. The range in gelatinization pasting temperature (PT) of the samples was 77.42°C–84.13°C. For microwave power of 480 W, PT was higher (84.13°C ± 0.16°C) than for untreated coarse cereals (77.42°C ± 0.40°C) (Singh et al. 2004). The final viscosity (FV) had a similar trend to PV and TV. The reduction of FV of microwave‐treated coarse cereals might be due to the formation of amylose‐lipid complexes, which led to a delay in the gelatinization and gelatinization pasting temperature (Hassan et al. 2021; Tarahi et al. 2022). The variation of coarse cereals breakdown (BD) under different microwave power indicated differences in thermal stability of starch. The low PV and BD values at 480 W indicated increasing heat resistance of coarse cereals, possibly due to the deformability of its starch particles (Fu et al. 2020; Reddy et al. 2017). For microwave power of 480 W, setback (SB) was 298.00 cP, which was lower than for the other conditions. The SB generally indicated the amylose leaching and rapid retrogradation of components in the starch paste (Fu et al. 2020). Microwave power significantly affected the gelatinization properties of coarse cereals, especially at 480 W, which may be related to the formation of starch–lipid complexes and the deformability of starch particles.

**TABLE 2:** RVA gelatinization characteristic values of coarse cereals after different microwave treatments.

Microwave power	Pasting Temp (°C)	Peak viscosity (cP)	Trough viscosity (cP)	Breakdown (cP)	Final viscosity (cP)	Setback (cP)
Untreated	77.42 ± 0.40^d^	1591.00 ± 5.20^a^	1548.67 ± 11.50^a^	36.00 ± 2.00^a^	2658.67 ± 6.81^a^	1112.00 ± 7.94^a^
160 W	78.49 ± 0.51^c^	1196.33 ± 8.33^b^	1194.33 ± 11.68^b^	27.00 ± 2.00^b^	2084.00 ± 4.58^b^	891.33 ± 6.66^b^
320 W	80.99 ± 0.11^b^	875.33 ± 6.03^e^	865.00 ± 12.49^e^	15.00 ± 1.00^d^	1269.33 ± 4.04^e^	409.00 ± 8.00^c^
480 W	84.13 ± 0.16^a^	696.00 ± 6.25^f^	683.67 ± 11.50^f^	22.33 ± 0.58^c^	972.33 ± 7.09^d^	298.00 ± 6.56^e^
640 W	81.61 ± 0.83^b^	910.33 ± 3.06^d^	887.00 ± 7.00^d^	25.67 ± 1.53^b^	1275.67 ± 6.11^d^	383.67 ± 4.16^d^
800 W	80.94 ± 0.06^b^	976.00 ± 7.00^c^	932.33 ± 16.04^c^	27.33 ± 0.58^b^	1329.67 ± 17.90^c^	394.00 ± 7.00^d^

*Note:* values with different letters in the same column are significantly different (*p* < 0.05).

### 3.6 Crystallization State of Microwave‐Treated Coarse Cereals

**FIGURE 4:**
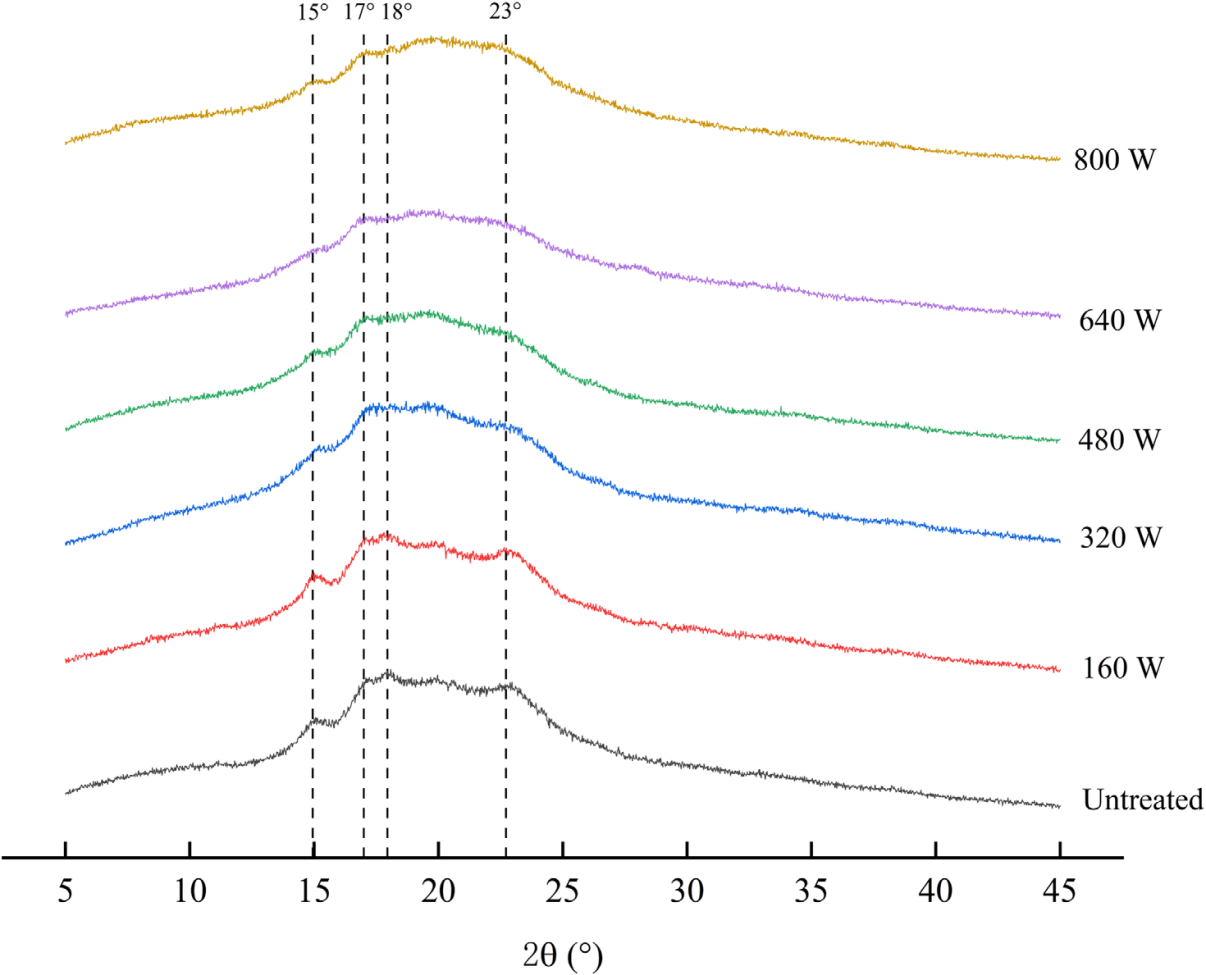
XRD patterns of coarse cereals after microwave treatment.

The XRD patterns of coarse cereals show A‐type crystallization mode for different microwave power conditions (Figure 4). The crystal structure of coarse cereals was destroyed by microwave treatment (Sharma et al. 2015). Both untreated and microwave‐treated coarse cereals exhibited typical polymorphic structures at 2θ near 15°, 17°, 18°, and 23° peaks. The intensity of the characteristic diffraction peaks of coarse cereals diminished after microwave treatment, suggesting that microwave treatment caused some disruption of the crystalline region of coarse cereals starch; however, it did not change the polymorphic structure as reported by Piecyk and Domian (2021).

### 3.7 Ordered Structure of Starch in Microwave‐Treated Coarse Cereals

**FIGURE 5:**
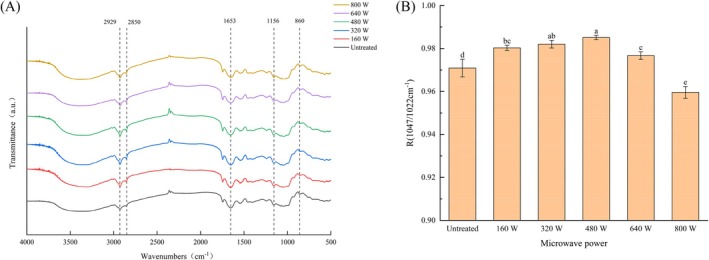
FTIR spectra (A) and peak strength ratio of 1047/1022 cm^−1^ (B) for coarse cereals after microwave treatment.

The FTIR spectra after different microwave treatments showed typical starch characteristic peaks of coarse cereals starch at 2929, 1156, and 860 cm^−1^ (Figure 5A), corresponding to the stretching vibration of –CH_2_ in starch, the vibration of glycosidic bond C–O–C, and the stretching deformation of C–H (Li et al. 2024). The absorption peak at 1653 cm^−1^ corresponds to the C=O stretching vibration of the protein amide I band (Basnet et al. 2016). The absorption peak intensity of 800 W at 1653 cm^−1^ was clearly greater than for 640, 480, 320, 160, and 0 W treatments, possibly due to protein denaturation and changes in protein content in coarse cereals starch. The peak at 2850 cm^−1^ is usually attributed to asymmetric C–H stretching in fat, which is due to a decrease in V‐shaped crystalline material and a decrease in amylose‐lipid complexes.

Infrared spectroscopy can be used to describe the changes of starch molecular sequence (Patel et al. 2017). The peak strength ratio of 1047/1022 cm^−1^ is regarded as the index of short‐range ordered structure of starch, with a higher ratio indicating better internal double‐helix order of starch (Flores‐Morales et al. 2012). Microwave treatment improved the short‐range ordered structure of starch (Figure 5B). To sum up, the starch particles in coarse cereals treated with 480 W had better ordered structure in terms of characteristic peaks; this is consistent with the report by Liu et al. (2024).

### 3.8 Microstructural Characteristics of Microwave‐Treated Coarse Cereals

**FIGURE 6:**
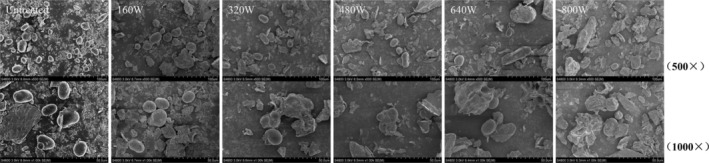
SEM images of microwave treated coarse cereals.

The surface morphology of coarse cereals after microwave treatment changed compared to untreated coarse cereals (Figure 6). With increased microwave power, the surface of starch particles became rough, and some cracks and holes appeared. The starch spheres of untreated coarse cereals were clearly defined, with a small amount of protein attached. After microwave treatment, a large number of fragments and irregular denatured proteins gradually appeared and were stuck together.

The higher the microwave power, the more serious the damage to starch in coarse cereals, and higher microwave power caused protein denaturation and starch gelatinization. Microwave pretreatment may result in adhesion between starch particles. This was mainly because starch particles absorb microwave energy and generate heat energy during pretreatment, resulting in water evaporation between particles and starch gelatinization (Zhang et al. 2021). When microwave power was increased, the heating speed was accelerated, and the water evaporation and gelatinization process between starch particles was also accelerated, making the adhesion phenomenon between particles more obvious.

### 3.9 Texture Characteristics of Cooked Coarse Cereals

Texture is the most important index to evaluate the eating quality of coarse cereals, which can be used to determine the palatability of cooked coarse cereals. The effects of microwave treatment power on the texture of cooked coarse cereals are shown in Table 3. The hardness of coarse cereals decreased after microwave treatment. When treated at 480 W for 10 min, coarse cereals' hardness reached 2039.15 ± 416.23 N. Microwave treatment can promote the gelatinization reaction of starch, making the coarse cereals softer and easier to digest. However, as microwave power increased to 800 W, coarse cereals' hardness decreased to 2721.13 ± 128.33 N. This was because the protein denaturation was excessive, which can cause the coarse cereals to lose their original taste and texture. There are no significant differences between the elasticity of microwave‐treated and untreated coarse cereals (Table 3). The range of elasticity was 0.24–0.39, which has little changed. Because the endosperm or cotyledon of coarse cereals produces elasticity, the endosperm or cotyledon was preserved after microwave treatment, so there was no difference in elasticity (Bai et al. 2021). With increased microwave power, the adhesiveness of coarse cereals increased slightly, and the difference became insignificant. Higher adhesiveness is not only related to starch but also to lipids and proteins inside coarse cereals. During microwave and cooking heat treatment, proteins and lipids easily form complexes with starch exuded from coarse cereals, which increases adhesiveness and affects quality characteristics. There is a negative correlation between adhesiveness and food chewability (Xing et al. 2023). The chewability of cooked coarse cereals decreased significantly with increased microwave power, indicating that microwaves did not destroy the original quality of coarse cereals but were beneficial to maintain original quality while decreasing hardness and improving adhesiveness. Therefore, microwave treatment at 480 W for 10 min had the best effect on improving the texture properties of cooked coarse cereals.

**TABLE 3:** Texture characteristics of cooked coarse cereals after different microwave treatments.

Microwave power	Hardness (*N*)	Elasticity	Cohesiveness	Adhesiveness (*N*)	Chewability (*N*)	Responsiveness
Untreated	3162.63 ± 165.75^a^	0.39 ± 0.08^a^	0.31 ± 0.02^b^	456.84 ± 60.04^e^	378.12 ± 57.27^a^	0.11 ± 0.05^a^
160 W	3026.31 ± 518.35^a^	0.29 ± 0.09^b^	0.30 ± 0.02^b^	626.42 ± 65.91^d^	258.80 ± 17.81^b^	0.11 ± 0.05^a^
320 W	2243.53 ± 357.36^c^	0.25 ± 0.07^c^	0.29 ± 0.03^b^	705.23 ± 38.61^c^	185.42 ± 43.35^c^	0.12 ± 0.02^a^
480 W	2039.15 ± 416.23^d^	0.24 ± 0.03^c^	0.27 ± 0.03^c^	761.92 ± 15.25^a^	133.13 ± 42.02^e^	0.13 ± 0.04^a^
640 W	2240.06 ± 132.8^c^	0.29 ± 0.12^b^	0.37 ± 0.04^a^	725.64 ± 78.59^b^	139.41 ± 28.57^e^	0.11 ± 0.02^a^
800 W	2721.13 ± 128.33^b^	0.31 ± 0.06^b^	0.39 ± 0.03^a^	703.01 ± 78.54^c^	146.63 ± 20.45^d^	0.08 ± 0.02^b^

*Note:* values with different letters in the same column are significantly different (*p* < 0.05).

### 3.10 Cooking Characteristics of Cooked Coarse Cereals

**FIGURE 7:**
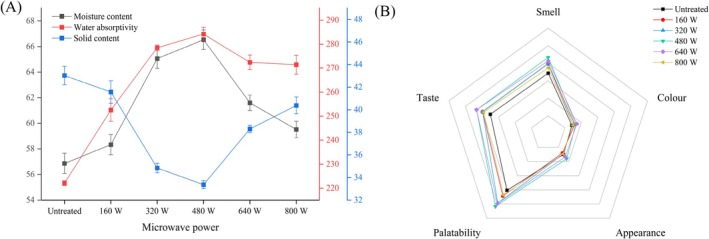
Cooking characteristics (A) and average radar map of sensory scores (B) of cooked coarse cereals after different microwave treatments.

Figure 7A shows the moisture content, water absorptivity, and solid content of coarse cereals for different microwave powers. After treatment at 480 W, the moisture of cooked coarse cereals increased significantly from 56.87% to 66.53% because microwaves can accelerate the movement of water molecules within the particles and promote the migration of water molecules (Wu et al. 2024). When treated with 640 W, the moisture content of cooked coarse cereals declined from 66.53% (480 W) to 61.60%. In the microwave process, water continued to heat up and escape from the intercellular space, leading to a decrease in moisture content.

With increased microwave power, water absorption of coarse cereals initially increased and then decreased. For a power of 480 W, the maximum water absorption was 284.15%, and water absorption increased by 62.08%. Under appropriate microwave power, cell structure and membrane permeability of cooked coarse cereals were improved, conducive to the infiltration and absorption of water molecules (An et al. 2024). The internal structure and water distribution of coarse cereals changed during microwave treatment. Microwave treatment may make the cell wall of coarse cereals more relaxed, which is conducive to water absorption (Wang et al. 2022). Therefore, in the initial stage of pretreatment, the water absorption of coarse cereals will increase. During the cooking process, with the gradual infiltration of water and the increase in temperature, the expansion and softening of coarse cereals and rice will occur. For low microwave power, water absorption increased due to the change in cell structure. However, for high microwave power pretreatment, the water may reach a saturated state, and some of the water may be squeezed out, resulting in reduced water absorption.

With the increase of microwave power, the solid content decreased gradually, and the lowest value (33.37%) was obtained for 480 W, which was 9.66% lower than for untreated coarse cereals. As microwave power continued to increase, the solid content also continued to increase, but at 800 W, the solid content was reduced by 2.65%. This may be due to the gelatinization of starch in coarse cereals during microwave treatment. The gelatinized starch is released after the starch particles absorb water and expand and break (Koriyama et al. 2017). Gelatinized starch has better fluidity and viscosity, which may cause part of the solid to dissolve or disperse in the gelatinized starch, thereby reducing the solid content. In the process of microwave pretreatment, some nutrients (fat and protein) in coarse cereals may dissolve in water, resulting in reduced solid content.

### 3.11 Sensory Evaluation Analysis

The taste characteristics of the cooked coarse cereals samples were described by the 10 sensory panelists. Compared with the untreated group, all microwave treatments showed significant improvements in sensory scores in a radar map (Figure 7B). The 480 W treatment significantly increased the sensory score of cooked coarse cereals to 95.17 points compared to 79.50 points for the control. The effects of other treatments on sensory scores were consistent with the instrument texture results (Figure 2). The improved smell and taste of cooked coarse cereals may be due to the vibration caused by absorption of microwave energy (Nayak and Mohapatra 2019) by some water molecules and flavor substances in the coarse cereals during the microwave treatment process, which helps the release and diffusion of flavor substances, thus allowing the coarse cereals to give off a rich aroma in the subsequent cooking process. In addition, microwave treatment can also promote the gelatinization of starch and the denaturation of protein in cereals, and these changes will also enhance the taste of coarse cereals. Microwave treatment can expand and gelatinize starch particles in coarse cereals (Figure 6), making them softer. At the same time, denaturation of protein under microwave action will also make the taste of coarse cereals more delicate, help to improve the palatability, reduce its hardness and chewability (Huang et al. 2021) (Table 2), and make it easier to chew and digest. During microwave treatment, the water distribution in grains was more uniform (Figure 3B). Microwave treatment can also promote the softening of cellulose in coarse cereals, making its texture more uniform and delicate, and making the appearance structure basically uniform. In summary, the above five sensory evaluation indicators were consistent with the measured textural properties, and the coarse cereals samples with microwave power of 480 W were praised in all aspects and had the highest total sensory score.

## 4 Conclusions

The effects of microwave treatment on coarse cereals quality were investigated from the aspects of texture, starch ordered structure, microscopic morphology, sensory score, and cooking quality. Microwave treatments could effectively improve the texture of coarse cereals. Among them, 480 W treatment had the most significant improvement, which not only decreased the hardness and chewability but also improved adhesiveness and sensory score of cooked coarse cereals. After 480 W treatment, part of the immobile water was transformed into tightly bound water. Furthermore, the relative crystallinity of starch in coarse cereals decreased. Microscopic morphology showed increased cracks and pores on the surface and cross section of coarse cereals treated by 480 W, which explained the improved coarse cereals quality. This study provided a green processing method for improving the eating quality of coarse cereals and investigated its improvement mechanism, which may facilitate the promotion of consumption of coarse cereals as a staple food, with a broad application prospect. The current study did not consider changes in nutritional quality of coarse cereals with microwave treatment, and this will be the focus of subsequent research.

## Author Contributions

**Hong Song:** conceptualization (equal), formal analysis (equal), funding acquisition (equal), investigation (equal), project administration (equal), resources (equal), supervision (equal), validation (equal), writing – review and editing (equal). **Dingyi Li:** data curation (equal), investigation (equal), methodology (equal), software (equal), validation (equal), visualization (equal), writing – original draft (equal). **Hongyu Zhang:** data curation (equal), methodology (equal), software (equal), visualization (equal). **Lina Yang:** investigation (equal), validation (equal). **Danshi Zhu:** formal analysis (equal), investigation (equal), supervision (equal). **Hongliang Fan:** formal analysis (equal), investigation (equal), validation (equal), visualization (equal). **Lu Han:** formal analysis (equal), investigation (equal), supervision (equal), validation (equal), visualization (equal). **Jinxin Li:** investigation (equal), supervision (equal), validation (equal), visualization (equal). **He Liu:** formal analysis (equal), funding acquisition (equal), project administration (equal), resources (equal), supervision (equal), validation (equal), visualization (equal).

## Conflicts of Interest

The authors declare no conflicts of interest.

## Data Availability

Data will be made available upon reasonable request.
